# Transforming growth factor β-activated kinase 1 transcriptionally suppresses hepatitis B virus replication

**DOI:** 10.1038/srep39901

**Published:** 2017-01-03

**Authors:** Jinke Pang, Geng Zhang, Yong Lin, Zhanglian Xie, Hongyan Liu, Libo Tang, Mengji Lu, Ran Yan, Haitao Guo, Jian Sun, Jinlin Hou, Xiaoyong Zhang

**Affiliations:** 1 State Key Laboratory of Organ Failure Research, Guangdong Provincial Key Laboratory of Viral Hepatitis Research, Department of Infectious Diseases, Nanfang Hospital, Southern Medical University, Guangzhou, China; 2 Institute of Virology, University Hospital of Essen, Essen, Germany; 3 Department of Infectious Diseases, Huashan Hospital, Fudan University, Shanghai, China; 4 Department of Microbiology and Immunology, Indiana University School of Medicine, USA

## Abstract

Hepatitis B Virus (HBV) replication in hepatocytes is restricted by the host innate immune system and related intracellular signaling pathways. Transforming growth factor β-activated kinase 1 (TAK1) is a key mediator of toll-like receptors and pro-inflammatory cytokine signaling pathways. Here, we report that silencing or inhibition of endogenous TAK1 in hepatoma cell lines leads to an upregulation of HBV replication, transcription, and antigen expression. In contrast, overexpression of TAK1 significantly suppresses HBV replication, while an enzymatically inactive form of TAK1 exerts no effect. By screening TAK1-associated signaling pathways with inhibitors and siRNAs, we found that the MAPK-JNK pathway was involved in TAK1-mediated HBV suppression. Moreover, TAK1 knockdown or JNK pathway inhibition induced the expression of farnesoid X receptor α, a transcription factor that upregulates HBV transcription. Finally, ectopic expression of TAK1 in a HBV hydrodynamic injection mouse model resulted in lower levels of HBV DNA and antigens in both liver and serum. In conclusion, our data suggest that TAK1 inhibits HBV primarily at viral transcription level through activation of MAPK-JNK pathway, thus TAK1 represents an intrinsic host restriction factor for HBV replication in hepatocytes.

Hepatitis B virus (HBV) infection is a major health concern worldwide, causing a wide spectrum of liver diseases in more than 240 million people. It is estimated that one million deaths occur each year due to HBV-related severe liver diseases, including liver cirrhosis, liver failure, and hepatocellular carcinoma (HCC)^1^. Currently, only nucleotide analogues and interferons (IFNs) are approved for the treatment of chronic hepatitis B (CHB) patients. However, it is difficult to achieve immune control or HBV clearance in the majority of patients using available antiviral agents^2^. It is generally accepted that host immune responses determine the clearance or persistence of HBV infection^3^.

HBV is a hepatotropic, enveloped virus of the *Hepadnaviridae* family with a partial double-stranded relaxed circular DNA genome. After infection of hepatocytes *via* the sodium taurocholate cotransporting polypeptide (NTCP) receptor^4^, the uncoated viral genome is transported to the nucleus and converted into an episomal covalently closed circular (ccc) DNA form, which serves as the template for synthesis of viral transcripts. The longest (3.5 -kb) mRNA includes two species, precore mRNA encodes the precore (HBeAg), and pregenomic RNA (pgRNA) translates core (HBcAg) and polymerase proteins. The pgRNA also serves as reverse transcription template after encapsidation into the HBcAg-derived nucleocapsid. Other transcripts including the 2.4 and 2.1 -kb viral mRNAs encode the large, middle, and small surface proteins (HBsAg), and the 0.7 -kb viral mRNA encodes the non-structural HBV X protein. The transcription of these viral mRNAs in hepatocytes is intensively controlled by the core, S1, S2, and X promoters and two enhancer regions (EnhI and EnhII)^5^. Many liver enriched transcription factors, such as hepatocyte nuclear factor 4α (HNF-4α) and farnesoid X receptor α (FXRα), are able to target the promoter and enhancer regions to regulate HBV replication and transcription^6^.

Efficient control of HBV infection requires coordinated action of both innate and adaptive immunity. Central to these antiviral responses is the secretion of IFNs or inflammatory cytokines, which promote specific T cell responses or target HBV-infected hepatocytes directly to limit virus infection^7^. HBV specific CD8+ T cells secrete IFN-γ and tumor necrosis factor-α (TNF-α) to clear HBV infection through a noncytopathic mechanism^8^. In primary human hepatocytes and human hepatoma cells, IFN-γ and TNF-α are able to inhibit HBV replication by inducing deamination and subsequent decay of cccDNA^9^. Moreover, toll-like receptor (TLR) ligands, as well as other cytokines (interleukin IL-1β, IL-6 and transforming growth factor-β [TGF-β], etc.), activate multiple downstream signaling pathways to suppress HBV replication in hepatoma cells through the transcriptional regulation of HBV RNA^10^,^11^,^12^,^13^. These studies suggest that TLRs or cytokines, which relate to innate and adaptive immunity, play an active role in the intracellular control of HBV replication and gene expression in hepatocytes.

TGF-β activated kinase 1 (TAK1, also known as MAP3K7) was discovered in 1995 as a serine/threonine kinase of the mitogen-activated protein kinase kinase kinase (MAPKKK) family^14^. It is a key adaptor protein in the signaling of TLRs and various cytokines, such as TNF-α, IL-1β and TGF-β^15^. Once activated by these cytokines or TLR ligands, TAK1 can be phosphorylated and polyubiquitinated by TNF-receptor-associated factors and in turn activates downstream pathways, including p38, c-jun N-terminal kinase (JNK) and IκB kinase. p38 and JNK of MAPK pathway control the transcription factor activator protein-1 (AP-1), while nuclear factor-kappa B (NF-κB) is activated by IκB kinase. In general, TAK1 regulates NF-κB and AP-1 activation in a cell-specific and receptor-specific manner^16^. Several reports by Seki’s group have addressed the function of TAK1 in liver development and proinflammatory signaling in hepatocytes. They found that hepatocyte-specific deletion of TAK1 in mice results in spontaneous hepatocyte death, inflammation, fibrosis, and carcinogenesis^17^,^18^.

Although TAK1 has been reported to be essential for cytokine signaling and cellular homeostasis in the liver, its role in chronic HBV infection remains largely unknown. Our previous reports indicate that TAK1 is involved in the suppression of HBV replication induced by the activation of TLRs in hepatocytes^11^. Therefore, it is of interest to study the potential function of TAK1, and the underlying mechanism, in the innate immune control of HBV replication.

## Results

### Silencing of TAK1 using siRNA, or treatment with TAK1 inhibitor (5-Z-7-Oxozeaenol), enhances HBV replication and gene expression in cell culture

**Figure 1:**
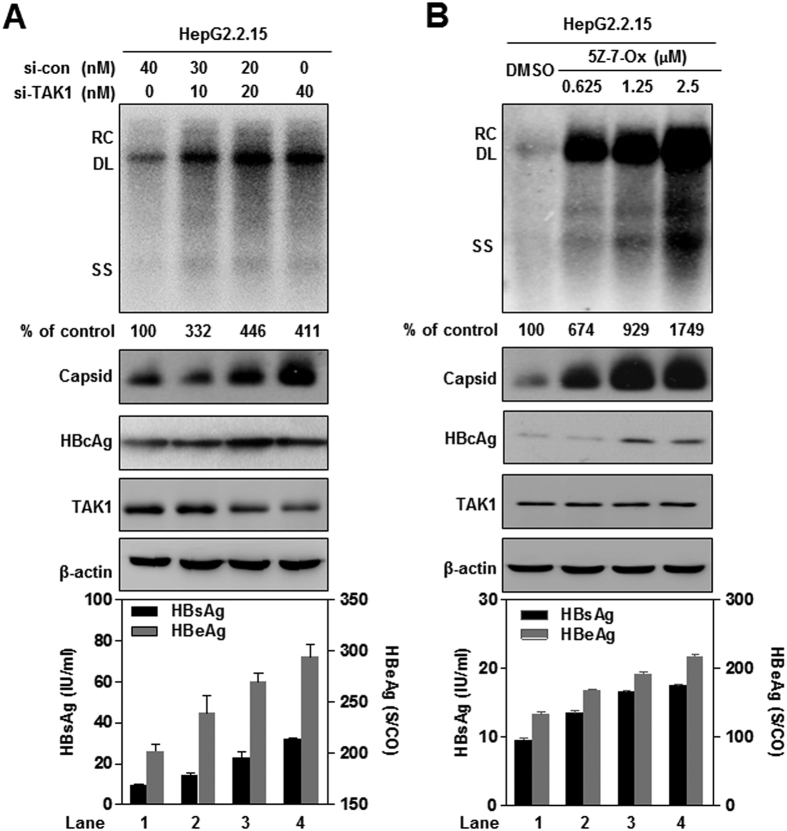
Silencing of TAK1 using siRNA, or treatment with TAK1 inhibitor (5-Z-7-Oxozeaenol), enhances HBV replication and gene expression in cell culture. (**A**) HepG2.2.15 cells were transfected with validated TAK1 siRNA or control siRNA, as indicated, and cultured for 4 days. Cell lysates and culture supernatants were harvested on day 4 after transfection, levels of HBV DNA replicative intermediates were determined by Southern blot analysis (top panel). TAK1, intracellular HBcAg expression and core particles were analyzed by Western blotting with β-actin as loading control (middle panel). HBsAg and HBeAg were detected in the culture supernatants by CMIA assay (bottom panel). (**B**) HepG2.2.15 cells were treated with the specific TAK1 inhibitor, 5-Z-7-Oxozeaenol (5Z-7-Ox), at the indicated concentrations for 4 days. Cell lysates and culture supernatants were harvested on day 4. Levels of HBV replicative intermediates (top panel), TAK1, intracellular HBcAg expression, core particles (middle panel), and levels of HBsAg and HBeAg (bottom panel) were determined as described above. RC, relaxed circular; DL, double stranded linear; SS, single stranded.

We first investigated the effects of TAK1 on HBV replication using loss-of function experiments. TAK1 specific (si-TAK1) or negative control (si-con) siRNAs were delivered into stably HBV-transfected HepG2.2.15 cells, and HBV replication and expression were evaluated. The silencing efficiency of si-TAK1 was confirmed by Western blot (Fig. 1A) and real-time PCR (Suppl. Fig. 1A). TAK1 knockdown dose dependently increased the amount of intracellular HBV replicative intermediates (HBV RI), HBV capsid, and HBcAg protein, as well as the titers of HBsAg and HBeAg in culture supernatants, compared to controls (Fig. 1A).

Similarly, co-transfection of siTAK1 into Huh7 cells with the replication-competent HBV plasmid, pHBV1.3, enhanced intracellular HBV replication and HBcAg expression, as well as HBsAg and HBeAg expression (Suppl. Fig. 1B). Furthermore, we confirmed the effect of TAK1 knock down on HBV transcription and replication in HBV infection system by using HepG2-NTCP cells. HepG2-NTCP cells with TAK1 stably knock down were generated by transducing cells with sh-TAK1 lentiviral vector, while sh-con containing non-silencing short hairpin RNA was used as a negative control. The knockdown efficiency of sh-TAK1 was verified by Western blot (Supp. Fig. 1C). As expected, knock down of TAK1 led to an increased level of both HBsAg and HBeAg on day 3, 6 and 9 post infection (Supp. Fig. 1D), which suggested that TAK1 knock down enhanced HBV replication and gene expression in infection system.

5Z-7-Oxozeaenol (5Z-7-Ox) is a natural resorcylic lactone of fungal origin that selectively inhibits TAK1 kinase activity, whereas it does not significantly inhibit TAK1 expression^19^. Consistently, treatment of HepG2.2.15 cells with 5Z-7-Ox had no significant effect on TAK1 expression, but led to an increase of intracellular HBV RI, HBV capsid, and HBcAg protein, as well as the titers of HBsAg and HBeAg in culture supernatants, in a dose-dependent manner (Fig. 1B). Thus, the kinase activity of TAK1 is required for TAK1-mediated inhibition of HBV replication and gene expression in hepatocytes.

### Overexpression of TAK1 inhibits HBV replication and gene expression in cell cultures

**Figure 2:**
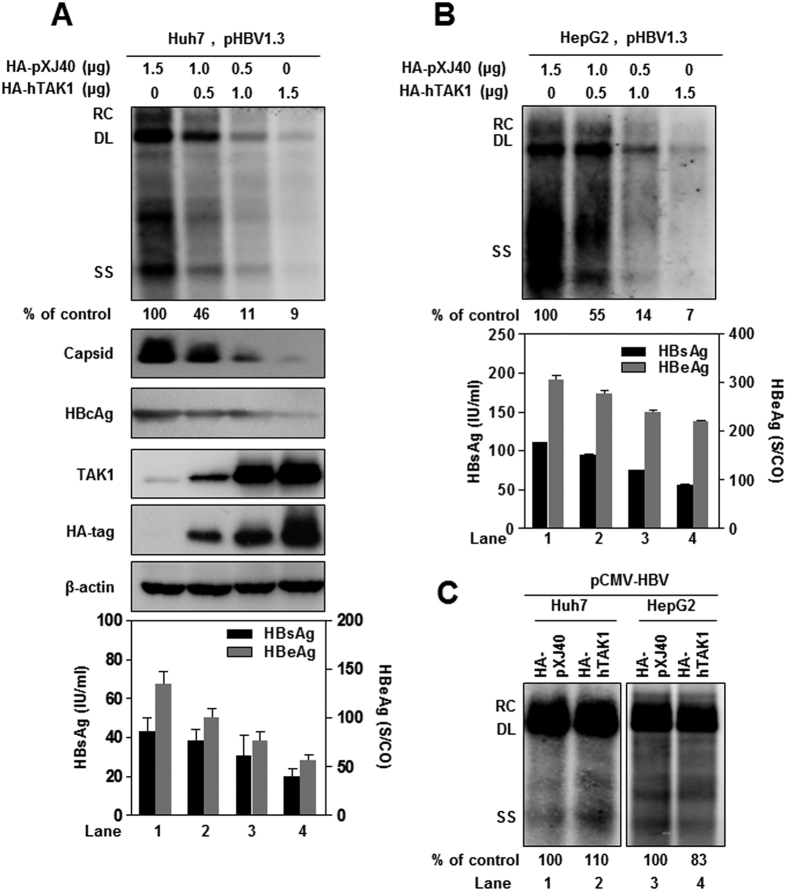
Overexpression of TAK1 inhibits HBV replication and gene expression in cell cultures. (**A**) A plasmid encoding pgRNA of wild-type HBV (pHBV1.3) was co-transfected into Huh7 cells with a control plasmid and a plasmid expressing TAK1 as indicated, and cultured for 3 days. Cells were harvested 72 h after transfection and the levels of HBV DNA replicative intermediates were determined by Southern blot analysis (top panel). HBV capsid, HBcAg, TAK1 and HA-tag protein levels were determined by Western blotting (middle panel; β-actin was used as a loading control), and the levels of HBsAg and HBeAg in supernatants were determined by CMIA. (**B**) HepG2 cells were transfected in the same way as Huh7 cells. HBV replicative intermediates and the HBV proteins HBsAg and HBeAg were determined as described above. (**C**) Huh7 and HepG2 cells were co-transfected with 1.5 μg of the CMV-HBV plasmid and 1.5 μg of pXJ40-HA or HA-hTAK1. The levels of HBV DNA replicative intermediates were determined by Southern blot. RC, relaxed circular; DL, double stranded linear; SS, single stranded.

To examine the effect of overexpression of TAK1 on HBV replication and gene expression, Huh7 cells were co-transfected with pHBV1.3 and either the plasmid encoding human TAK1 (HA-hTAK1) or the control vector (HA-pXJ40). As shown in Fig. 2A, overexpression of TAK1 significantly reduced the amount of intracellular HBV RI, HBV capsid, and HBcAg protein, as well as the titers of HBsAg and HBeAg in culture supernatants, in a dose-dependent manner. The expression of TAK1 protein in transfected cells was confirmed by TAK1 and HA-tag Western blot analysis (Fig. 2A, middle panel). Consistently, overexpression of TAK1 also inhibited HBV replication and gene expression in HepG2 cells transfected with pHBV1.3 (Fig. 2B). However, TAK1 (HA-TAK1) did not significantly inhibit HBV replication when it was co-transfected into Huh7 or HepG2 cells with pCMV-HBV, in which HBV pgRNA transcription is under the control of the cytomegalovirus-IE promoter (Fig. 2C). This observation suggests that TAK1-mediated antiviral activity against HBV was limited to HBV transcription governed by the HBV core promoter.

### TAK1 regulates HBV core promoter activation through FXRα to suppress HBV transcription

**Figure 3:**
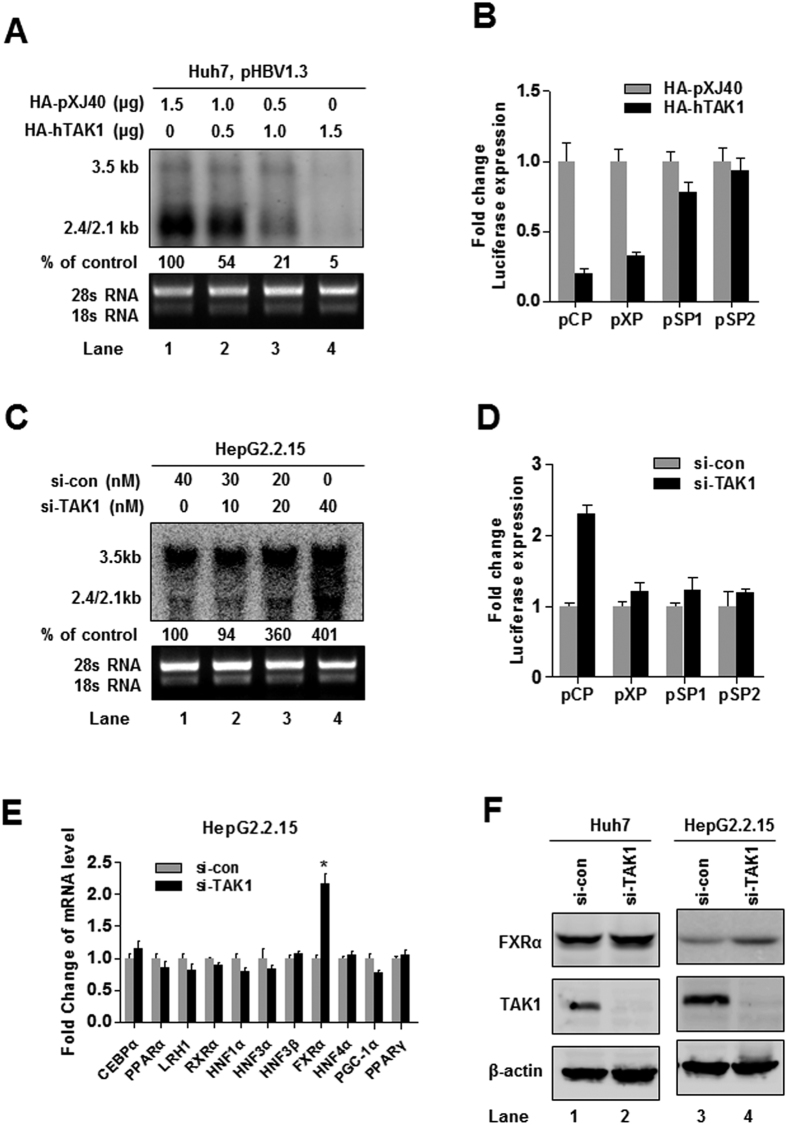
TAK1 regulates HBV core promoter activation through FXRα to suppress HBV transcription. (**A**) Huh7 cells were co-transfected with pHBV1.3 and HA-hTAK1 and control vector (HA-pXJ40) as indicated. Levels of HBV RNAs were determined by Northern blot 72 h after transfection. (**B**) Huh7 cells were co-transfected with 100 ng each of four reporter plasmids containing HBV promoters (pCP, pXP, pSP1, and pSP2) and 100 ng of HA-TAK1 or pXJ40-HA to analyze the effect of TAK1 overexpression on transcription activity from HBV promoters. For each transfection, 100 ng of pRL-TK was included as an internal control for transfection efficiency. Cells were harvested 48 h after transfection, and firefly and Renilla luciferase activities were measured; luciferase levels were normalized against those generated by the pGL3-basic control plasmid. (**C**) HepG2.2.15 cells were transfected with siRNA targeting TAK1 (si-TAK1) or control siRNA (si-con) as indicated. Cells were harvested 72 h after transfection and total cellular RNA were extracted and analyzed by Northern blot. (**D**) Huh7 cells were co-transfected with 100 ng of each of four reporter plasmids containing HBV promoters (pCP, pXP, pSP1, and pSP2) and 20 nM of si-TAK1 or negative control siRNA. For each transfection, 100 ng of pRL-TK was included as an internal control of transfection efficiency. Cells were harvested 48 h after transfection, and firefly and Renilla luciferase activities were measured and normalized against those produced by the pGL3-basic control plasmid. (**E**) HepG2.2.15 cells were transfected with si-TAK1 or control siRNA (20 nM). The mRNA levels of associated transcription factors were determined by RT-PCR. (**F**) HepG2.2.15 cells or Huh7 cells were transfected with si-TAK1 or control siRNA (20 nM) and FXRα and TAK1 expression were verified by western blot.

Considering that the hepatocyte-specific deletion of TAK1 in mice results in spontaneous hepatocyte death and carcinogenesis^17^,^18^, we performed CCK-8 assay to examine whether TAK1 overexpression or inhibition influence cell viability. The results showed that TAK1 plasmid, siTAK1 and 5Z-7Ox had no significant effect on HepG2.2.15 and Huh7 cell viability compared to controls (Supp. Fig. 2A,B,C). In order to verify whether TAK1-mediated inhibition of HBV replication and gene expression was due to transcriptional regulation, we examined the effect of TAK1 on HBV RNA levels and the transcription activity of HBV promoters. Northern blot analysis showed a decrease of HBV transcripts in Huh7 cells under TAK1 overexpression (Fig. 3A), with core promoter (CP) and X promoter (XP) transcriptional activities reduced to approximately 20% and 30%, respectively (Fig. 3B). In contrast, knockdown of TAK1 by si-TAK1 increased HBV transcripts (Fig. 3C) and CP activity approximately 2.5-fold, but had no significant effect on other (XP, SP1, and SP2) promoters (Fig. 3D). Since HBV core promoter transcription activity is controlled by a set of liver-specific transcription factors^6^, we examined whether TAK1 affects the expression of these transcription factors. Eleven transcription factors which have previously been reported to bind to the HBV core promoter were selected and screened by real-time PCR. Interestingly, we observed that the expression of FXRα was elevated by approximately 2.0-fold after knockdown of TAK1 in HepG2.2.15 cells (Fig. 3E), the upregulation of FXRα by TAK1 knockdown was further verified by Western blot (Fig. 3F). Transcription activity of the FXRα promoter was also enhanced after silencing of TAK1, indicating that TAK1 negatively regulates FXRα expression at the level of transcription (Suppl. Fig. 2D).

### TAK1 inhibition of HBV replication and gene expression requires activation of downstream MAPK pathways

**Figure 4:**
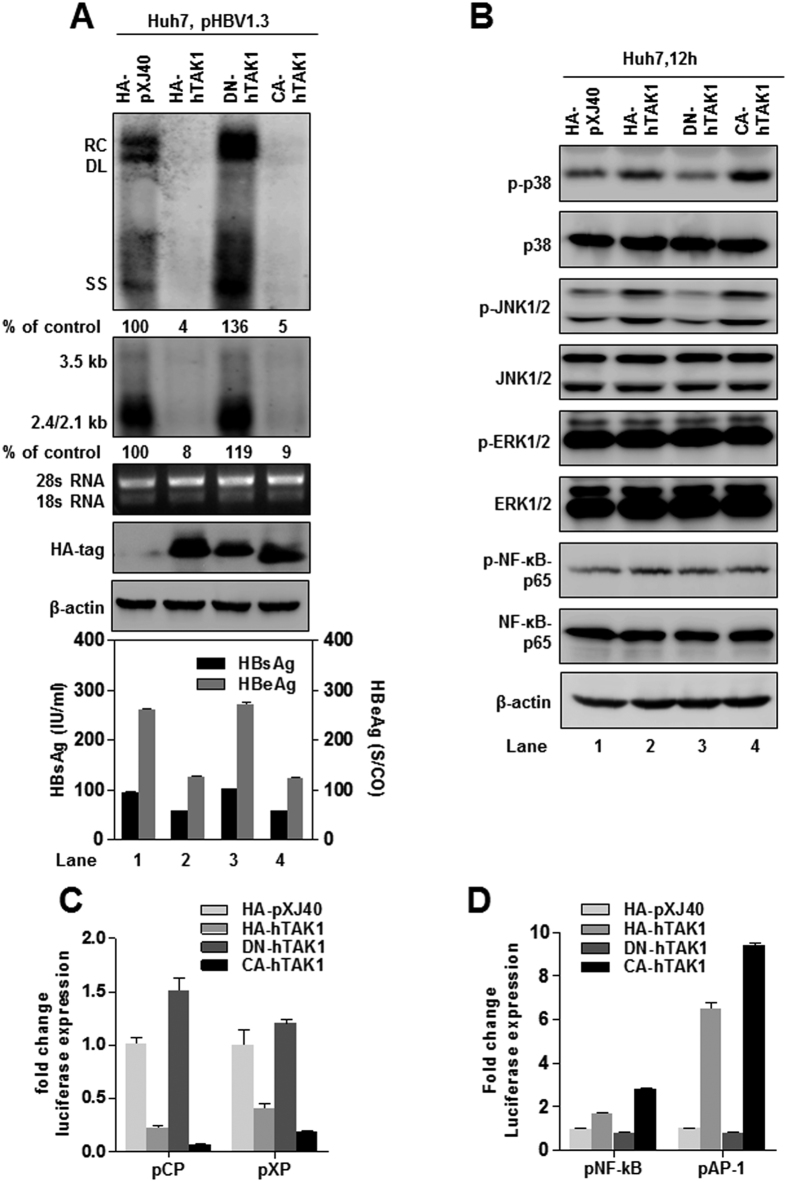
TAK1 inhibition of HBV replication and gene expression requires activation of downstream MAPK pathways. (**A**) Huh7 cells were co-transfected with 1.5 μg of pHBV1.3 and 1.5 μg of pXJ40-HA, or plasmids expressing HA-hTAK1, DN-hTAK1 or CA-hTAK1. Cells were harvested 72 h after transfection. HBV RI, viral RNAs and HBsAg and HBeAg were determined as described above. HA-tag and β-actin (loading control) were examined by Western blot, 28 S/18 S rRNAs served as loading controls for Northern blot analysis. (**B**) Huh7 cells were co-transfected with 100 ng of pCP or pXP, and 100 ng of pXJ40-HA or plasmids expressing HA-hTAK1, DN-TAK1 or CA-TAK1. Cells were harvested 48 h after transfection and firefly and Renilla luciferase activities were measured and normalized against those generated by the pGL3-basic control plasmid. (**C**) Huh7 cells were transfected with 1.5 μg of pXJ40-HA or plasmids expressing HA-hTAK1, DN-TAK1, and CA-TAK1. Phosphorylation and basal expression levels of p38, JNK, ERK, and NF-κBp 65 were analyzed by Western blot 12 h after transfection, using β-actin as loading control. (**D**) Huh7 cells were seeded in 24-well plates and transfected with 100 ng of the reporter plasmids pNF-κB-luc (pNF-kB) or pAP1-luc (pAP-1) and 100 ng of pXJ40-HA, hTAK1, DN-TAK1, or CA-TAK1. For each transfection, 100 ng of pRL-TK was included as an internal control of transfection efficiency. Cells were harvested 48 h after transfection and firefly and Renilla luciferase activities were measured and normalized against those generated by the pGL3-basic control plasmid.

TAK1 can be activated by multiple cytokines, and a TAK1 deletion mutant missing the N-terminal 22 amino acids is constitutively active^20^. In addition, TAK1 containing a point mutation in its ATP-binding domain (K63W), which abolishes its kinase activity, is unable to activate downstream signaling pathways^20^. We constructed dominant negative (DN-hTAK1) and constitutively active (CA-hTAK1) TAK1 plasmids as described^14^. Similar to wild type HA-hTAK1, transfection of CA-hTAK1 suppressed HBV replication, transcription, and gene expression in Huh7 cells. (Fig. 4A, lanes 2 and 4). In contrast, a small increase in HBV replication was observed by DN-hTAK1 transfection, perhaps due to its dominant-negative effect on the endogenous TAK1 activity (Fig. 4A, lane 3). Consistently, CA-hTAK1 also suppressed transcription from HBV core and X promoters (Fig. 4B). Similar to the observations in Huh7 cells, DN-hTAK1 did not affect HBV replication and gene expression in HepG2 cells (suppl. Fig. 3A). Consistent with the kinase inhibitor results (Fig. 1B), these results further confirmed that TAK1 inhibition of HBV replication depends on its kinase activation of downstream pathways.

To explore which signaling pathways were activated by TAK1, different types of TAK1 expression plasmids were delivered into Huh7 cells, which were then cultured for 12 h. Western blotting analysis of total cell lysates was performed to detect phosphorylated p38, JNK1/2, ERK1/2, and NF-κB p65. The results showed that both HA-hTAK1 and CA-hTAK1 increased the phosphorylation of p38 and JNK1/2, and, to a lesser extent, that of NF-κB p65. As expected, DN-hTAK1 had no effect on the level of phosphorylation of these proteins (Fig. 4C). No differences were observed in basal protein expression levels of ERK, JNK, p38, and NF-κB p65, or phosphorylation of ERK1/2 in any transfections (Fig. 4C). We also confirmed that inhibition of TAK1 activity by 5Z-7-Ox treatment, or si-TAK1 transfection, led to a significant suppression of the phosphorylation of MAPK JNK/ERK/p38 proteins in Huh7 cells (Suppl. Fig. 3B,C).

In addition, luciferase assays of the reporter plasmids pNF-κB and pAP-1 confirmed that overexpression of HA-hTAK1 and CA-hTAK1 led to a significant increase of NF-κB and AP-1 promoter activities in Huh7 cells; however, DN-TAK1 had a negligible effect on luciferase expression from these reporters (Fig. 4D). Moreover, the induction of AP-1 transcription activity by TAK1 was stronger than that of NF-κB, suggesting that TAK1 may preferentially activate downstream MAPK p38 and JNK pathways in hepatocytes.

### The MAPK-JNK pathway is involved in TAK1-mediated antiviral function

**Figure 5:**
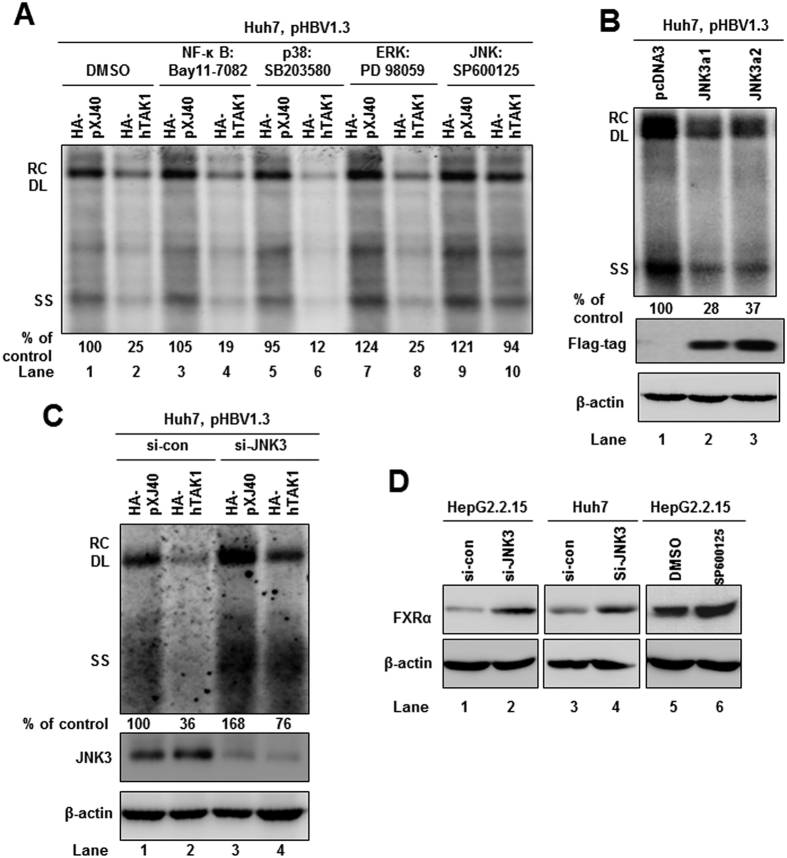
The MAPK-JNK pathway is involved in TAK1-mediated antiviral function. (**A**) Huh7 cells were co-transfected with 1.5 μg of pHBV1.3 and 1.5 μg of HA-hTAK1 or pXJ40-HA plasmids and, 6 h after transfection, cells were left untreated (lane 1, 2) or treated with 2 μM of different inhibitors for 3 days as indicated. The dimethyl sulfoxide concentration in all experimental groups was 0.1%. HBV RI was analyzed by Southern blot 72 h after transfection. (**B**) Huh7 cells were co-transfected with 1.5 μg pHBV1.3 and 1.5 μg pcDNA3, or plasmids expressing JNK3a1 or JNK3a2, which were expressed for 3 days. HBV DNA replication was analyzed by Southern blot 72 h after transfection. Flag-tag and β-actin were examined by Western blot. (**C**) Huh7 cells were co-transfected with 1.5 μg of pHBV1.3 and 1.5 μg of HA-hTAK1 or pXJ40-HA plasmids, plus 20 nM of negative control siRNA or si-JNK3, as indicated. HBV DNA replication was analyzed by Southern blot 72 h after transfection. JNK3 and β-actin were examined by Western blot. (**D**) HepG2.2.15 or Huh7 cells were transfected with 20 nM of si-JNK3 or treated with 2 μM of SP600125 for 48 h, and FXRα expression was determined by Western blot, with β-actin used as a loading control. *p < 0.05.

To identify which downstream signaling pathway is essential in the TAK1-mediated suppression of HBV replication in hepatocytes, inhibitors of the NF-κB pathway and the different arms of the MAPK pathway, were used to determine whether they abolish the suppressive effect of TAK1 on HBV replication. The specific inhibitory effects of the different signaling inhibitors were validated by Western blot, as previously described^21^. The MAPK-JNK pathway inhibitor SP600125 clearly blocked the antiviral effect of TAK1 (Fig. 5A, lane 10), indicating the essential role of this pathway in controlling of HBV replication. Of interest, the p38 pathway inhibitor, SB203580, enhanced the antiviral effect of TAK1 on HBV replication (Fig. 5A, lane 6), consistent with a previous publication indicating that suppression of MAPK-p38 inhibits hepatitis B virus replication in human hepatoma cells^22^.

Furthermore, we investigated which molecules of the JNK pathway are involved in controlling HBV replication. Proteins, including JIP1, JIP2, JIP3, JIP4, KSR, POSH, MKK7, JNK1, JNK2, and JNK3, are reported to be members of the JNK signaling pathway^23^. By screening with specific siRNAs targeting these molecules in HepG2.2.15 and Huh7 cells, we identified that silencing of JNK3 could enhance HBV replication, compared with negative control siRNA (Suppl. Fig. 4A,B). In contrast, overexpression of JNK3a1 or JNK3a2 in Huh7 cells inhibited HBV replication (Fig. 5B). Moreover, the inhibitory effect on HBV replication by TAK1 could be partially blocked by co-transfection of si-JNK3 with HA-TAK1 (Fig. 5C). Interestingly, we observed that silencing of JNK3, or treatment with the JNK pathway inhibitor, SP600125, could up-regulate the expression of FXRα in Huh7 and HepG2.2.15 cells (Fig. 5D). These results suggest that JNK3 may also regulate FXRα expression to regulate HBV replication.

### Administration of TAK1 by hydrodynamic injection led to HBV inhibition in a mouse model of chronic HBV infection

**Figure 6:**
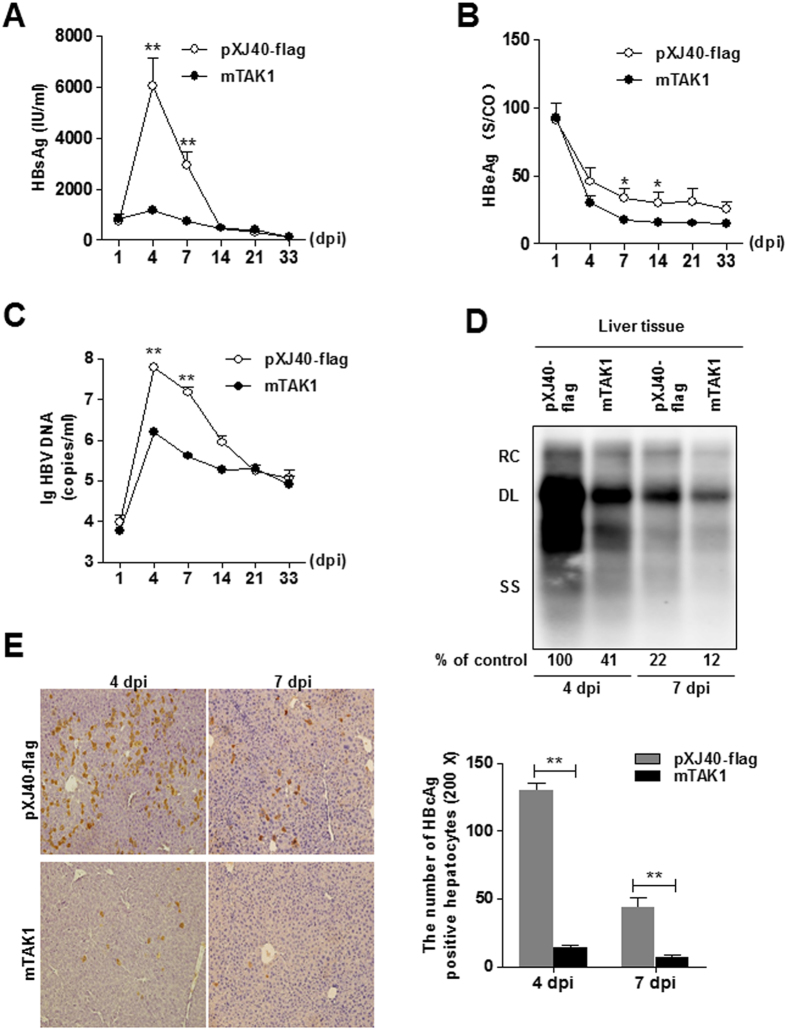
Administration of TAK1 by hydrodynamic injection led to HBV inhibition in a mouse model of chronic HBV infection. Six-week old male C57BL/6 mice were co-injected with 10 μg of pAAV-HBV1.2 and 10 μg of flag-mTAK1 (n = 8) or control plasmid (n = 8) by hydrodynamic injection. Serum and liver samples were collected at different time points. (**A**,**B**) Serological markers of HBV infection (HBsAg, HBeAg) were assayed at the indicated time points. (**C**) Serological HBV DNA was assayed at the indicated time points. Data are presented as log_10_ copies/ml. (**D**) Mouse liver tissues were collected from the control and TAK1 treated mice at 4 and 7 dpi, and HBV RI levels were determined by Southern blot. RC, relaxed circular; DL, double stranded linear; SS, single stranded. (**E**) Liver tissue sections obtained at 4 and 7 dpi were stained with anti-HBc antibodies (magnification, 200x, left panel). The number of HBcAg positive hepatocytes was counted, and data are presented as means ± SEM (right panel). *p < 0.05, **p < 0.01.

To further investigate the role of TAK1 in HBV infection *in vivo*, we induced chronic HBV infection in wild-type C57BL/6 mice using hydrodynamic injection (HI) to deliver the HBV replicative plasmid. pAAV-HBV1.2 plasmid (10 μg) was injected hydrodynamically into the tail veins of 6 week-old male C57BL/6 mice, along with 10 μg of either a plasmid expressing mouse TAK1 (mTAK1), or a control plasmid (pXJ40-flag). Serum and liver samples were collected at different time points as indicated. HBsAg levels in the sera of the mTAK1 plasmid injection group were significantly lower at 4 and 7 dpi than those in the pXJ40-flag group (*p* = 0.0047 and 0.0003, respectively; Fig. 6A). In addition, levels of HBeAg were significantly lower at 7 (*p* = 0.0135) and 14 (p = 0.0379) dpi (Fig. 6B). Serological HBV DNA was significantly inhibited in the mTAK1 group compared with the control group at 4 (p = 0.0002) and 7 (p = 0.0002) dpi (Fig. 6C). Consistent with changes in serological DNA and HBV antigens, Southern blot analysis showed that liver HBV RI was inhibited in the mTAK1 group, compared with the control group, at 4 and 7 dpi (Fig. 6D). By IHC staining, HBcAg expression and the frequency of HBcAg-positive hepatocytes were lower in the mTAK1 injection group than in controls (Fig. 6E). These findings suggest that TAK1 inhibits HBV replication and gene expression *in vivo*.

### Increased TAK1 expression in the liver tissues of chronic HBV infection with hepatic inflammation

CHB patients in the immune active status exhibit liver inflammation over a period of months or years, as the immune system attempts to clear the infection, resulting in abnormal ALT levels and fluctuant viral loads^24^. To examine the role of TAK1 in chronic HBV infection, we enrolled 27 treatment-naïve CHB patients and divided these patients into “Inflammatory” group (n = 9) and “Non-inflammatory” group (n = 18) (Suppl. Fig. 5A). By analyzing the levels of TAK1 mRNA in liver biopsy samples obtained from these CHB patients, we found that “Inflammatory” group had significantly elevated levels of TAK1 mRNA expression in the liver, compared with “Non-inflammatory” group (Suppl. Fig. 5B). However, there was no significant correlation of TAK1 expression and HBV DNA levels, indicating that multiple factors (host and viral) are involved in shaping HBV fitness and immunopathogenesis. Considering the observed antiviral effect of TAK1 in hepatocytes, we speculated that the upregulation of TAK1 in the inflammation liver might participate in the immune control of HBV replication in CHB patients.

## Discussion

In this study, to better define the mechanism whereby host factors restrain HBV, we investigated the impact of TAK1 and its downstream pathways on HBV replication and gene expression. We found that TAK1 suppresses HBV replication in HBV-producing cell lines, as well as in a hydrodynamic injection mouse model. Moreover, TAK1 exerts its antiviral effect at the level of transcription by regulation of FXRα expression, and its effects depended on intracellular signal transduction. We also identified JNK3, a component in the TAK1-activated MAPK-JNK pathway, as a potential downstream factor responsible for suppression of HBV. Therefore, we propose that TAK1 is an intrinsic host antiviral factor that restricts HBV amplification in hepatocytes.

The pathogenesis of chronic HBV infection involves complicated mechanisms related to viral persistence and the host immune system^3^. Although HBV is unable to modify host cellular gene transcription significantly, or induce IFN-stimulated gene expression in the liver of experimentally infected chimpanzees^25^, approximately 50% of HBV patients produce detectable levels of serum TNF-α, IL-6, and IL-1β within 10 days after initiation of viral expansion and before the peak of viremia^26^. These induced cytokines exhibit antiviral activity in both HBV transgenic mice and cell culture systems^8^,^27^. Of note, in response to these cytokines, TAK1 mediates the activation of downstream signaling pathways as an important adaptor protein^15^. Our findings clearly demonstrate that TAK1 suppresses HBV replication and gene expression in cell lines and a mouse model, which may explain the role of these cytokines in the intracellular antiviral response of hepatocytes. Further, by analyzing the levels of TAK1 mRNA in liver biopsy samples obtained from CHB patients, we found that inflammatory group patients with abnormal ALT levels had significantly elevated levels of TAK1 mRNA expression than non-inflammatory group (Fig. 6). Thus, we speculate that the upregulation of TAK1 might contribute to viral control in chronic HBV infection.

In addition to the cytokines mentioned above, TLR-mediated innate immunity also plays an important role in control of HBV replication^28^. Previously, we have determined that TLR2 or TLR4 ligands are able to inhibit HBV and Woodchuck hepatitis virus replication through activation of IFN-independent pathways in primary woodchuck hepatocytes or hepatoma cell lines^11^,^21^. Moreover, direct activation of cellular pathways through the expression of signaling adaptors also inhibited HBV replication. Guo *et al*. showed that the activation of the NF-κB pathway by expression of TLR adaptor molecules (MyD88, TRIF, or IPS-1) in hepatoma cells suppresses HBV replication and transcription^29^. TAK1 can be activated by TLR ligands and is necessary for TLR-mediated signal transduction and production of antiviral cytokines^15^. However, we found that treatment of HepG2.2.15 cells using conditioned medium from cells transfected with a plasmid expressing TAK1, did not reduce HBV replication or gene expression (data not shown). Our observations of the antiviral function of TAK1 thus reemphasize the importance of the TLR signaling pathway in the control of HBV replication, and indicate that the antiviral activity of TAK1 against HBV may be largely dependent on intracellular factors, rather than the secreted cytokines.

To identify intracellular factors involved in TAK1-mediated antiviral effects, we first determined the downstream events in the NF-κB pathway, and different arms of the MAPK pathway, mediated by TAK1 over-expression. Using various forms of TAK1 expression plasmids, we found that TAK1 induced signal transduction was necessary for HBV inhibition. Interestingly, the P38 and JNK pathways, but not the ERK or NF-κB pathways, were predominantly activated by wild type and constitutively active TAK1 in hepatocytes. Furthermore, the JNK pathway was apparently responsible for the suppression of HBV replication, as the JNK inhibitor, SP600125, and JNK3 siRNA attenuated the antiviral effects of TAK1. Based on these observations, we conclude that TAK1 may trigger JNK pathway activation, leading to reduction of HBV replication and transcription.

Previously, both our group and Chang *et al*. demonstrated that suppression of phosphorylation of p38 using the inhibitor SB203580 could impede intracellular HBV replication^11^,^22^, while another study reported that activation of the p38 pathway could enhance HBV replication and gene expression^30^. As the inhibition of the p38 pathway by SB203580 has been shown to enhance TAK1-induced activation of the JNK pathway^31^, it is likely that TAK1 might preferentially activate the JNK pathway to override the positive effect of the p38 pathway on HBV replication. By screening of siRNAs, we identified JNK3 as a downstream transmitter of TAK1-mediated antiviral signaling. These new findings suggest that the components of the JNK pathway should be analyzed further to investigate their role(s) in control of HBV replication.

The data presented herein further elucidate the mechanisms involved in HBV transcription regulation. There are many liver-enriched transcription factors, especially nuclear receptors, which can bind to HBV promoters to regulate HBV transcription and to facilitate its replication^6^. Previously, the cytokine IL-6, which activates TAK1, was found to inhibit HBV replication at the level of transcription and down-regulate expression of HNF1α and HNF4α^12^. To determine whether the observed reduction of HBV RNAs upon overexpression of TAK1 is *via* the mechanism of transcriptional regulation, we determined that HBV pgRNA can be efficiently reduced by TAK1 in an HBV core promoter-dependent manner. We found that TAK1 knockdown, as well as JNK pathway inhibition, could upregulate the mRNA and protein expression of FXRα, a transcription factor that binds to two motifs within the HBV enhancer II and core promoter regions to enhance HBV transcription and replication^32^,^33^. However, due to the limitations of real-time RT-PCR screening, there may be other undefined transcription factors regulated by TAK1, as we observed a discrepancy in the results of TAK1 over-expression and knockdown on X promoter activity. Nevertheless, our data suggest that the activation of JNK pathway by TAK1 leads to downregulation of transcription factor FXRα and in turn results in HBV suppression. Although FXRα activation by bile acids antagonizes the JNK signaling pathway in liver carcinogenesis^34^, the mechanism of JNK pathway regulation of FXRα requires further investigation.

Taken together, our results provide insights into a new role for the TAK1/JNK/FXRα cascade in regulating HBV replication and gene expression. The TAK1-mediated antiviral effect can be induced by cytokines or activation of TLRs, which results in non-cytopathic inhibition of HBV replication in hepatocytes, an important process in immune control of HBV infection. Non-cytopathic inhibition of viral gene expression is an attractive means of therapeutic control for infections, and for HBV in particular. Future advances in the understanding of intracellular anti-HBV host factors will assist in the development of strategies to find a cure for HBV infection.

## Materials and Methods

### Reagents

The TAK1 inhibitor 5Z-7-Oxozeaenol and the NF-κB pathway inhibitor BAY11–7082 were purchased from Sigma-Aldrich (St. Louis, MO, USA). SP600125 (JNK pathway inhibitor), SB203580 (p38 pathway inhibitor), and PD98059 (ERK pathway inhibitor) were purchased from Cell Signaling Technology (Danvers, MA, USA). Pre-designed small interfering RNAs (siRNAs) were bought from Qiagen (Hilden, Germany) and their sequences are listed in Supplementary Table 1.

### Plasmids and vector construction

The HBV (genotype D, subtype ayw) replication-competent plasmids, pHBV1.3 and pCMV-HBV, in which the transcription of viral pgRNA is governed by an authentic HBV core promoter and a cytomegalovirus-IE promoter, respectively, have been described previously^35^. To construct the HA-tagged human TAK1 (HA-hTAK1) and flag-tagged murine TAK1 (flag-mTAK1) vectors, the full-length coding regions for hTAK1 and mTAK1 were PCR amplified and cloned into empty pXJ40-HA or pXJ40-flag vectors (Addgene, Cambridge, MA, USA). Plasmids containing constitutively active hTAK1 (CA-hTAK1), which lacks the first 66 nucleotides, cloned into pXJ40-HA and dominant negative hTAK1 (DN-hTAK1), with a point mutation of lysine-63 in the ATP-binding site, were constructed according to a previous report^20^, using a QuickChange site-directed mutagenesis kit (Stratagene, Santa Clara, CA, USA). pcDNA3-flag-JNK3a1 (13758#) and pcDNA3-flag-JNK3a2 (13759#) were purchased from Addgene (Cambridge, MA, USA). Luciferase reporter vectors pNF-κB-luc, pAP-1-luc, and pRL-TK were purchased from Clontech (Mountain View, CA, USA). The pGL3-basic derived luciferase reporter vectors, pSP1, pSP2, pCP, pXP, and pFXRα were previously generated and described^33^. All constructed vectors were subjected to DNA sequencing to verify that the nucleotide sequences were correct. The primers (Invitrogen, Shanghai, China) used for cloning are listed in Supplementary Table 2.

### Cell culture and transfection

Human hepatoma cell lines, HepG2, Huh7 and HepG2-NTCP cells stably expressing human NTCP (kindly provided by Prof. Wenhui Li)^4^ were grown in Dulbecco’s modified Eagle’s medium supplemented with 10% fetal bovine serum, 100 U/ml penicillin, and 100 μg/ml streptomycin, and maintained at 37 °C in 5% CO2. HepG2.2.15 cells with integrated dimers of the HBV genome (GenBank Accession number U95551) were cultured with 500 μg/ml of G418 (Sigma-Aldrich). Plasmids or siRNAs were transfected into cells at the indicated concentrations using Lipofectamine 2000 (Life Technologies, Carlsbad, CA, USA) according to the manufacturer’s instructions.

### Lentivirus transduction and HBV infection

The GV248 lentiviral vector sh-TAK1 containing short hairpin RNA targeting sequence (5′- GTGTGTCTTGTGATGGAAT-3′) and the negative control lentiviral vector sh-Con were obtained from Genechem Company Ltd (Shanghai, China) and were used to knock down TAK1 expression. To generate clones stably knock down TAK1, the HepG2-NTCP cells first were infected at a multiplicity of infection of 20 with sh-TAK1 or sh-Con. Stable clones were selected after 2 weeks using puromycin and the expression level of TAK1 was determined by Western blot. HBV infection of HepG2-NTCP cells was performed according to Prof. Wenhui Li’s protocol^4^. Briefly, HepG2-NTCP cells were seeded in 6-well plates and infected with HBV at 100 genome equivalent (GEq)/cell in the present of 4% polyethylene glycol (PEG 8000; Sigma) for 16 h, and then rinsed three times with phosphate-buffered saline and maintained in the maintenance medium containing 2.5% dimethyl sulfoxide. The supernatant samples were collected at 3, 6 and 9 days post infection and HBsAg and HBeAg were assessed.

### Analysis of HBV replication and gene expression

HBV replicative intermediates (HBV RI) from intracellular core particles, and total cellular RNA were extracted from cells or liver tissue as described previously^33^. Samples were electrophoresed into agarose gels and blotted onto Hybond-XL membranes (GE Healthcare, Marlborough, MA, USA). Then, the membranes were probed with a DIG-labeled specific full-length HBV riboprobe, generated using the PCR DIG Probe Synthesis Kit (Roche, Basel, Switzerland), and visualized with CDP-Star using an ImageQuant LAS 2000 mini system (GE Healthcare). The densitometry of nucleic acid bands were analyzed by Image-Pro Plus software (Media Cybernetics Inc, Silver Spring, MD) and the values were presented below each of the blots as the percentage of control samples. The levels of HBsAg and HBeAg in culture supernatants were determined using the Architect system and HBsAg and HBeAg CMIA kits (Abbott Laboratories, North Chicago, IL, USA) according to the manufacturer’s instructions.

### Western blot analysis

Protein samples were subjected to SDS-PAGE gel electrophoresis and blotted with primary antibodies recognizing HA-tag, TAK1, β-actin, JNK1/2/3, ERK1/2, p38, NF-κB p65, phospho-JNK1/2, phospho-ERK1/2, phospho-p38, phospho-NF-κB (p65) (Cell Signaling Technology), FXRα (R&D Systems, Minneapolis, MN, USA), and HBcAg (Abcam, Cambridge, MA, USA). Protein bands were visualized using ECL Plus western blotting detection reagents (Amersham Biosciences, Buckinghamshire, UK) and an ImageQuant LAS 2000 mini system, as previously described^11^.

### Quantitative realtime-PCR assay

DNase I-treated total cellular RNA was reverse-transcribed into cDNA using a QuantiTect Reverse Transcription Kit (Qiagen). The expression of different cellular genes was determined by quantification of specific mRNAs using commercial QuantiTect Primer Assays (Qiagen; primer sequences are not available). Real-time PCR was performed using a SYBR Green Master kit (Roche) in the Roche LightCycler 480 system. For each sample, RT-PCR was performed in triplicate. The expression levels of each gene are presented as values normalized against 10^6^ β-actin transcripts.

### Luciferase reporter assay

Approximately 2 × 10^5^ HepG2.2.15 or Huh7 cells were seeded in 24-well plates one day before transfection. siRNA (20 nM) or vectors expressing hTAK1 (100 ng) were co-transfected with various firefly reporters (100 ng), and a Renilla luciferase reporter pRL-TK (100 ng) was added to each transfection to correct for transfection efficiency. After 48 h, cells were washed with PBS and resuspended in lysis buffer from the DualGlo Luciferase Assay system (Promega, Madison, WI, USA), followed by detection of luciferase activity in a luminometer (PerkinElmer, Norwalk, CT, USA). Relative firefly luciferase activities were normalized to those of Renilla luciferase activities.

### Cell viability analysis

HepG2.2.15 cells transfected with si-TAK1 or treated with 5Z-7-Oxozeaenol and Huh7 cells transfected with HA-hTAK1 plasmid were seeded at a density of 2 × 10^3^ cells per well in 96-well plates, and then the viability of the cells was assessed from 4 replicates at 3 or 4 days post transfection using the Cell Counting Kit-8 (Dojindo, Kumamoto, Japan).

### Microarray analysis of intrahepatic gene expression in CHB patients

A total of 27 treatment-naïve CHB patients were enrolled in this study and liver biopsies were collected. All patients were recruited with informed written consent. The study was approved by the Institutional Ethics Committee for human studies at Huashan Hospital, Fudan University. All clinical investigations have been conducted according to the principles expressed in the Declaration of Helsinki. Total mRNA was isolated from liver specimens and the gene expression profiling was performed by using Affymetrix Human U133 Plus 2 arrays at Ebioservice, Inc (Shanghai, China). Microarray data were analyzed using GeneSpring GX 10 (Agilent). The complete microarray dataset will be published separately. In this study, the TAK1 gene expression data were expressed as log_2_ values with mean ± SD, a *p*-value < 0.05 (t-test) is considered significant.

### Animal experiments

C57BL/6 mice (male, 5–6 weeks old) were treated according to the guidelines of the National Institutes of Health for Animal Care and Use. The animal experimental protocol was approved by the Nanfang hospital animal ethic committee. Hydrodynamic injection was carried out as described previously^36^. Briefly, pAAV-HBV1.2 plasmid (10 μg; kindly provided by Professor Pei-Jer Chen, National Taiwan University), together with 10 μg of flag-mTAK1 or control plasmid (pXJ40-flag), were co-injected into the tail vein of mice in a volume of saline equivalent to 10% of the body mass of the mouse. The total volume was delivered in 5–8 seconds. Mice negative for HBsAg 1 day post injection (dpi) were excluded from the experiments. Eight mice were included per group. Serum samples were collected from mice at the indicated time points for HBsAg, HBeAg, and HBV DNA testing. Liver tissue samples were also collected and HBV replication was examined by Southern blot. Immunohistochemistry (IHC) *staining* were performed on formalin-fixed and paraffin-embedded 4 μM liver tissue sections. Sections were incubated overnight at 4 °C with appropriate concentrations of rabbit anti-HBc, and then incubated with the Dako Chemate Envision Kit (Dako, Shanghai, China). The reaction was visualized using CheMate 3–3′-diaminobenzadine plus chromogen, and the number of HBc-positive cells in the liver sections was independently counted by two blinded observers based on four high-power microscopic fields (400×).

### Statistical analyses

Statistical analyses were carried out using GraphPad Prism 5.0 software (San Diego, CA, USA). Analysis of variance with Student’s t-test was used to determine significant differences in multiple comparisons. The Mann–Whitney *U* test was used for two-group comparisons. P values < 0.05 were considered statistically significant. Representative data from a series of at least three experiments are shown. Data are presented as means ± standard error of the mean (SEM).

## Additional Information

**How to cite this article**: Pang, J. *et al*. Transforming growth factor β-activated kinase 1 transcriptionally suppresses hepatitis B virus replication. *Sci. Rep.* **7**, 39901; doi: 10.1038/srep39901 (2017).

**Publisher's note:** Springer Nature remains neutral with regard to jurisdictional claims in published maps and institutional affiliations.

## Supplementary Material

Supplementary Information
