# Supplementary material for: Transforming growth factor β-activated kinase 1 transcriptionally suppresses hepatitis B virus replication
Source: Sci Rep. 2017 Jan 3;7:39901. doi: 10.1038/srep39901 (PMC5206675; doi:10.1038/srep39901)
Supplement: Supplementary Information [file srep39901-s1.pdf]

Supplementary information to:

Transforming growth factor β-activated kinase 1 transcriptionally suppresses hepatitis B virus replication

Jinke Pang, Geng Zhang, Yong Lin, Zhanglian Xie, Hongyan Liu, Libo Tang, Mengji Lu, Ran Yan, Haitao Guo, Jian Sun, Jinlin Hou, Xiaoyong Zhang*

Table of contents:

Supplemental Tables

Supplemental Table 1--2
Supplemental Table 2--3

Supplemental Figures

Suppl. Fig. 1--- 4
Suppl. Fig. 2--- 5
Suppl. Fig. 3--7
Suppl. Fig. 4--8
Suppl. Fig. 5--9
Suppl. Fig. 6---10
Suppl. Fig. 7---11
Suppl. Fig. 8---12
Suppl. Fig. 9---13
Suppl. Fig. 10---14
Suppl. Fig. 11---15

Supplemental Tables

Supplemental Table 1 siRNAs oligonucleotide sequences

Table 1. siRNA oligonucleotide sequences

Gene name	Cat. No.	Oligonucleotide sequences
**JIP1 (MAPK8IP1)**	SI02223949	CTGGAGGAGTTTGAGGATGAA
**JIP2 (MAPK8IP2)**	SI00300790	GACTTCCAGGAGTTTGAGATG
**JIP3 (MAPK8IP3)**	SI00109683	CAGCCGCAACATGGAAGTACA
**JIP4**	SI04191677	ATGGAGCGAAACCAGTATAAA
**POSH**	SI04157531	TTCCTGGTATAAAGTTGGTTA
**KSR1**	SI02650361	CAAGACGTCTCTGGACATCAA
**MKK4**	SI00301749	AATTGGACGAGGAGCTTATGG
**MKK7**	SI00300720	AAAGATGACAGTGGCGATTGT
**JNK1 (MAPK8)**	SI00300783	AAGAAGCTAAGCCGACCATTT
**JNK2 (MAPK9)**	SI00300797	AAGAGAGCTTATCGTGAACTT
**JNK3 (MAPK10)**	SI02222934	TCCGAGCACAATAAACTCAAA
**TAK1 (MAP3K7)**	SI00300741	AAAGCGTTTATTGTAGAGCTT

All the siRNAs were purchased from Qiagen Company (Hilden, Germany). The siRNA nonspecific negative control (siR-con) were purchased from Dharmacon (siR-con, siGENOME Non-Targeting siRNA #2; Cat. D-001210-02. Lafayette, CO).

Supplemental Table 2. Primers used for real time PCR and luciferase reporter cloning

Gene name	Application	Type	Sequence 5’-3’	Position of 5’-base
**HBV DNA**	**real time PCR**	**forward**	5′-GTTGCCCGTTTGTCCTCTAATTC-3′	465
		**reverse**	5′-GGAGGGATACATAGAGGTTCCTT-3′	563
**human TAK1**	**hTAK1 cloning**	**forward**	5′-CGCGGATCC ATGTCTACAGCCTC-3′	419
		**reverse**	5′-CGGGGTACC TCATGAAGTGCCT-3′	2158
**mouse TAK1**	**mTAK1 cloning**	**forward**	5′-CGCGGATCC ATGTCGACAGCCTCCGCC-3′	157
		**reverse**	5′-CGGGGTACC TCATGAAGTGCCTTGTCGTT-3′	1977
**DN-hTAK1**	**Dominant negative**	**forward**	5′- CGACGCGTCTCACTTTTGGAAGAGAAAC-3′	580
	**hTAK1 cloning**	**reverse**	5′-CCTAGATCTCTTATATAATATACCCGC-3′	620
**CA-hTAK1**	**Constitutive active**	**forward**	5′-CGCGGATCC ATGTCCCAGGTCCTCAACTTT-3′	485
	**hTAK1 cloning**	**reverse**	5′- CGGGGTACC TCATGAAGTGCCTTGTCG-3′	2158

The underlined parts of primers indicate the specific cleavage sites of restriction enzymes (“_”, BamHI; “_ _”, KpnI;).

GenBank accession numbers: HBV, V01460; human TAK1, NM_003188.3; mouse TAK1, NM_009316.1

Supplemental figure legends

Suppl. Fig. 1.

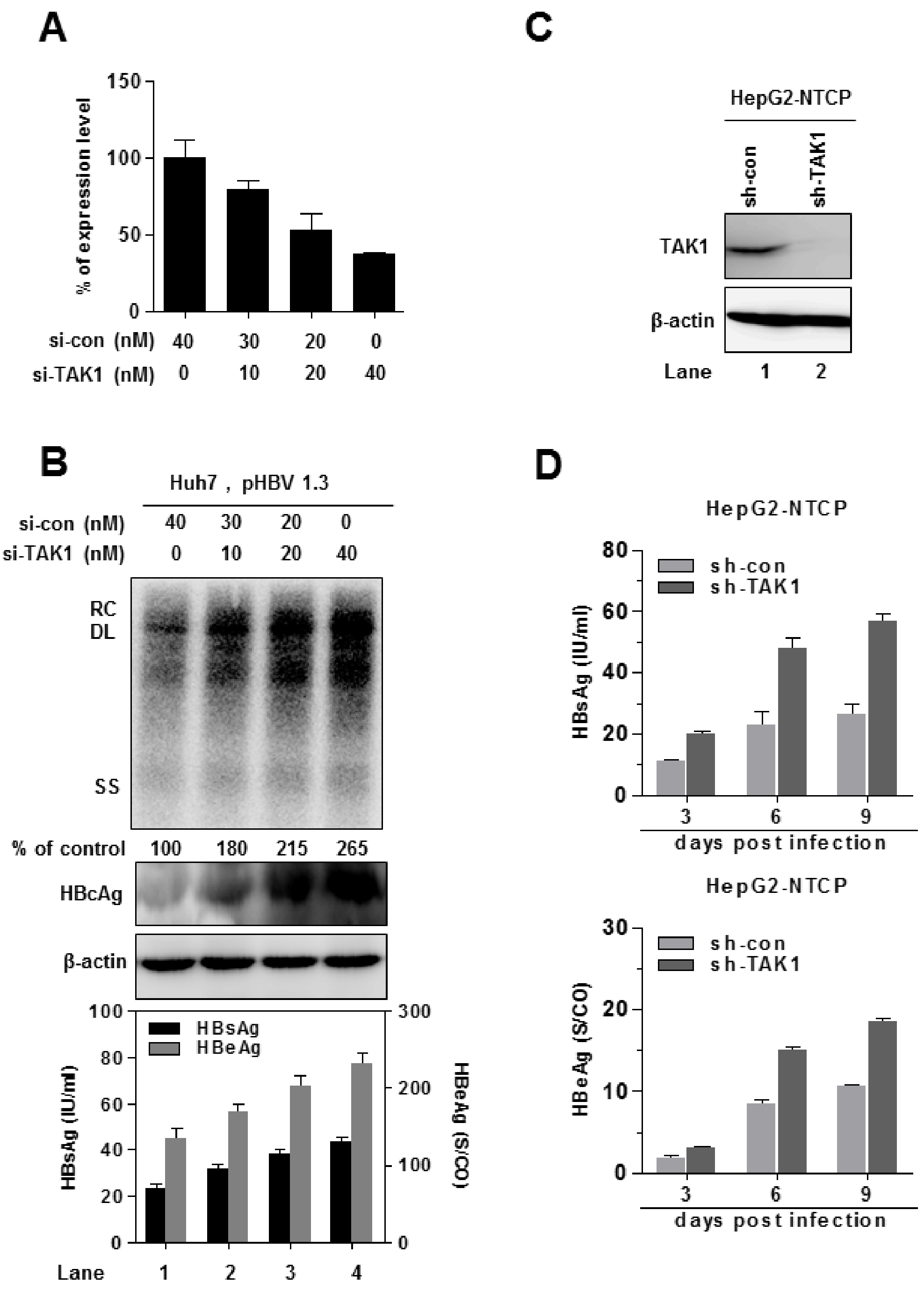

Suppl. Fig. 1. Knockdown efficiency of si-TAK1 and its effect on HBV replication in huh7 and HepG2-NTCP cells. (A) HepG2.2.15 cells were transfected with validated TAK1 siRNA or negative control siRNA at the indicated concentrations and cultured for 4 days. The mRNA level of TAK1 was determined by real-time RT-PCR. (B) A HBV replicative plasmid, pHBV1.3, was co-transfected into Huh7 cells with

si-TAK1 or negative control siRNA and HBV RI and HBcAg levels were determined by Southern blot (upper panel. RC, relaxed circular; DL, double stranded linear; SS, single stranded) and western blot (middle panel), respectively. The levels of HBsAg and HBeAg were examined by CMIA. β-actin served as a loading control. (C) HepG2-NTCP cells were infected at a multiplicity of infection of 20 with sh-TAK1 or sh-Con. Stable clones were selected after 2 weeks using puromycin and the expression level of TAK1 was determined by Western blot. (D) HBV infection of HepG2-NTCP cells was performed and the supernatant samples were collected at 3, 6 and 9 days post infection and HBsAg and HBeAg were assessed.

Suppl. Fig. 2.

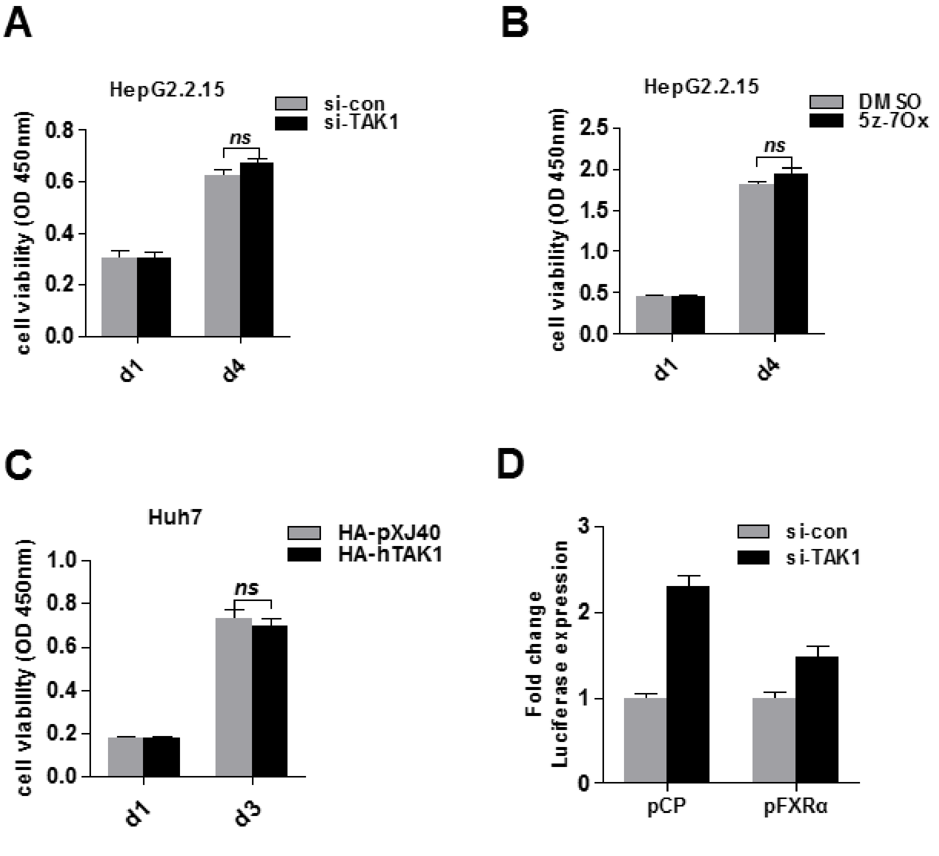

Suppl. Fig. 2. The effect of TAK1 overexpression or inhibition on HepG2.2.15 cells and Huh7 cells viability (A) HepG2.2.15 cells were transfected with TAK1 siRNA or negative control siRNA at 20nM and cultured for 4 days. Cells viability was determined using CCK-8 assay at day 1 and day 4. (B) HepG2.2.15 cells were treated with 5Z-7-Ox or DMSO control at 2.5 μM and cultured for 4 days. Cells viability was

determined using CCK-8 assay at day 1 and day 4. (C) Huh7 cells were transfected with HA-TAK1 plasmid or HA-pXJ40 control at 1.5 μg/ml and cultured for 3 days. Cells viability was determined using CCK-8 assay at day 1 and day 3. (D) Firefly luciferase reporters (pFXRα or pCP; 100 ng) and a Renilla luciferase reporter (TK-pRL; 100 ng) were transfected into Huh7 cells. Luciferase activity was measured after co-transfection with 20 nM of si-TAK1 or negative control siRNA for 48 h and normalized against levels in mock transfected cells.

Suppl. Fig. 3.

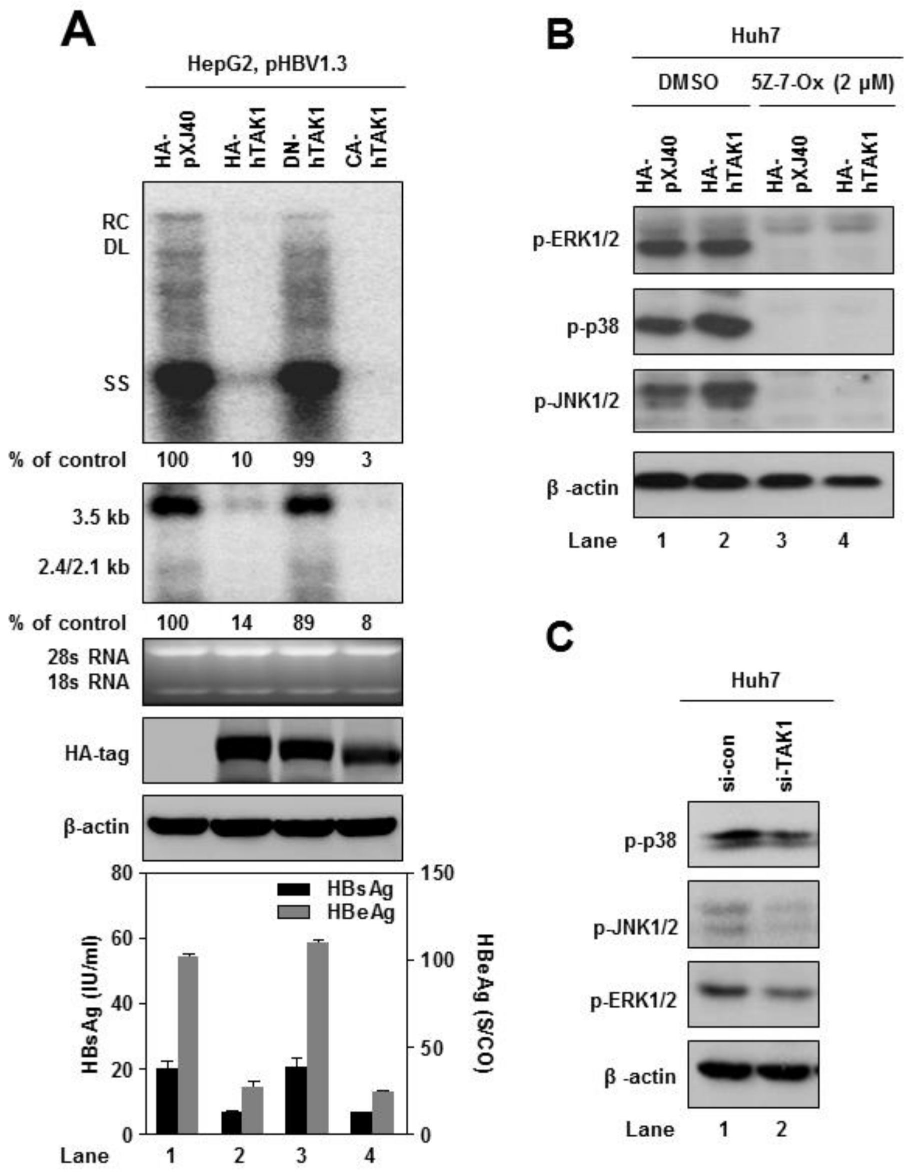

Suppl. Fig. 3. The effects of different forms of TAK1 on HBV replication and of si-TAK1 and 5-Z-7-Ox on the MAPK signaling pathway. (A) HepG2 cells were co-transfected with 1.5 μg of pHBV1.3 and 1.5 μg of pXJ40-HA or plasmids expressing HA-hTAK1, DN-hTAK1, or CA-hTAK1. Levels of HBV RI and viral RNAs were determined by Southern and northern blot analyses, respectively. Levels of HBsAg and HBeAg in the cell supernatants were determined by CMIA. (B) Huh7 cells were transfected with 1.5 μg HA-TAK1 or pXJ40-HA. Cells were treated with 2 μM of 5-Z-7-Oxozeaenol (5Z-7-Ox) or DMSO (control) as indicated, 6 h after transfection, for 2 days. Cells were harvested for detection of phosphorylated ERK,

p38, and JNK. (C) Huh7 cells were transfected with si-TAK1 or negative control siRNA at a concentration of 20 nM. Cells were harvested 72 h after transfection for detection of phosphorylated ERK, p38, JNK.

Suppl. Fig. 4.

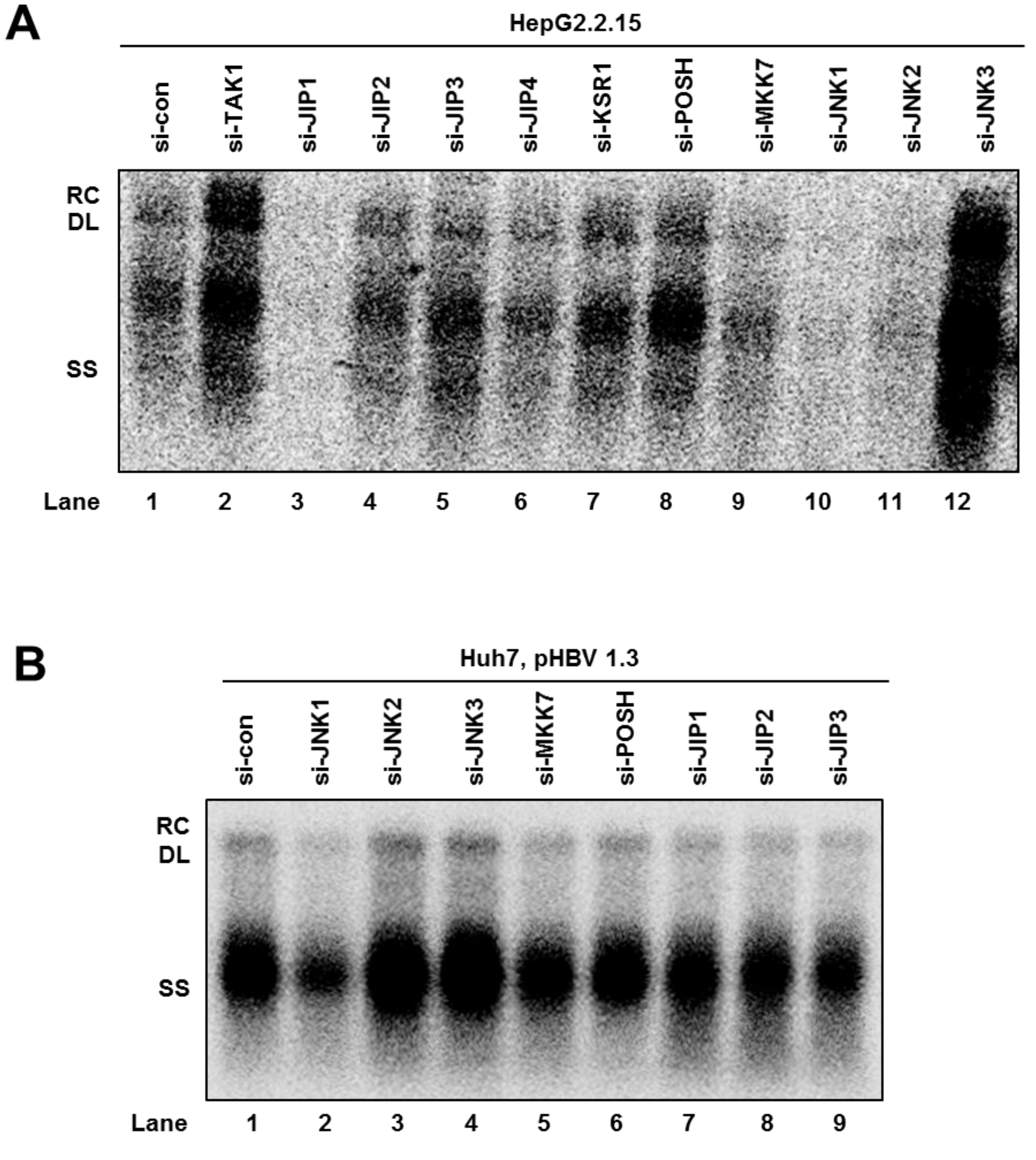

Suppl. Fig. 4. Screening of adaptor proteins of JNK pathway involved in control of HBV replication. (A) HepG2.2.15 cells were co-transfected with siRNAs (20 nM) targeting JNK pathway transcripts. Cells were harvested 72 h after transfection and levels of HBV RI determined by Southern blot analysis. (B) Huh7 cells were co-transfected with 1.5 μg pHBV1.3 and siRNAs (20 nM) targeting JNK pathway

transcripts . Cells were harvested 72 h after transfection and HBV RI levels detected by Southern blot analysis.

Suppl. Fig. 5.

A

Group	Non-inflammatory	Inflammatory
No. of patients	18	9
Age (year)	38.72(24-58)	40.22(33-52)
Sex (male/female)	13/5	6/3
ALT (ULN)	0.6(0.2-0.9)	6.9(2.6-20.1)
HBV DNA titer (log_{10} copies/ml)	5.3(2.7-8.6)	6.6(5.7-7.7)
HBeAg/Anti-HBe	9/9	9/0

B

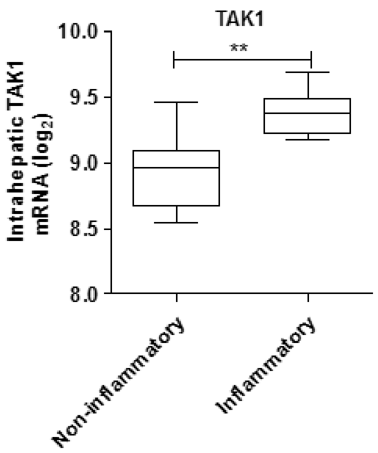

Suppl. Fig. 5. Intrahepatic mRNA levels of TAK1 in CHB patients. (A) A total of 27 treatment-na ïve CHB patients were enrolled and divided into “Inflammatory” group and “Non-inflammatory” group. In each group, values are expressed as average (range) or number.（B）Liver biopsies were obtained from these patients and total mRNA was isolated from liver specimens and gene expression profiling was performed using Affymetrix Human U133 Plus 2 arrays at Ebioservice, Inc (Shanghai, China). Microarray data were analyzed using GeneSpring GX 10 (Agilent). TAK1 gene expression data are expressed as log2 values with mean ± SD using GraphPad Prism 5.0 software. Upper limits of normal (ULN); Alanine transaminase (ALT); Hepatitis B e antigen (HBeAg); n.s.: not significant. **： $p < 0.01$.

Suppl. Fig. 6. Full-length gels of Figure 1

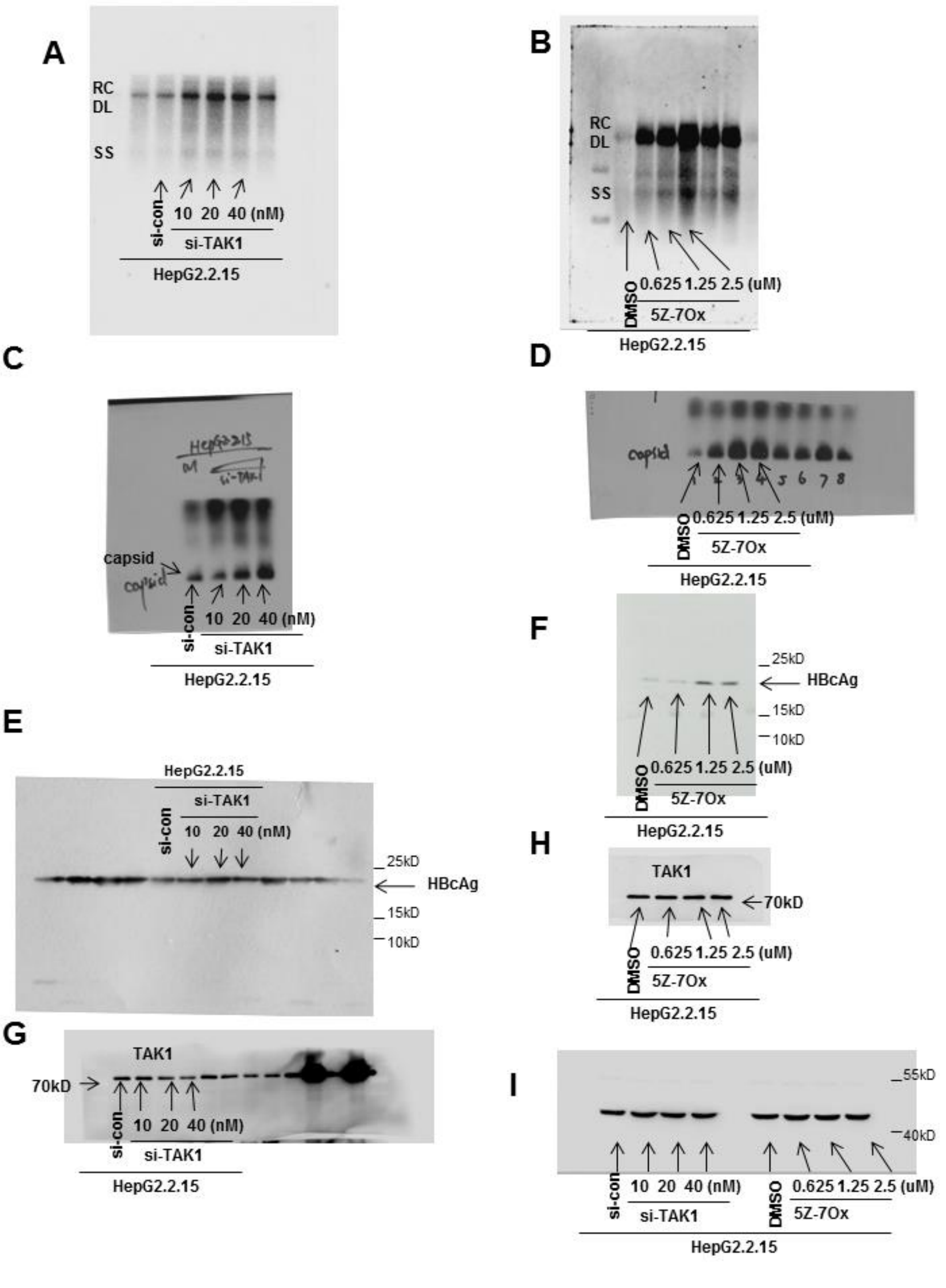

(A, C, E, G, H, I) Full-length gels of Fig. 1A. (B, D, F, H, I) Full-length gels of Fig. 1B.

Suppl. Fig. 7. Full-length gels of Figure 2

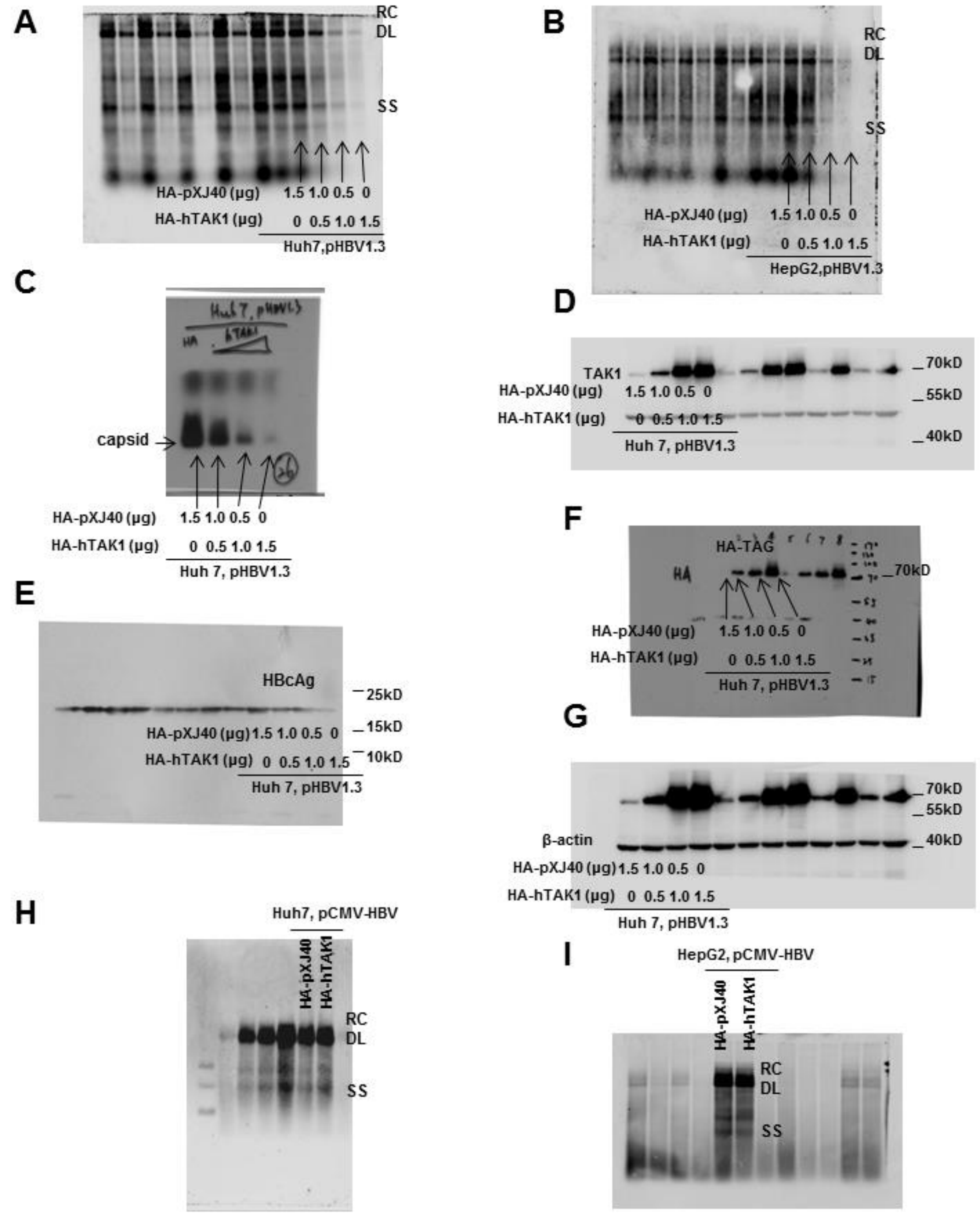

(A, C, E, D, F, G) Full-length gels of Fig. 2A. (B) Full-length gels of Fig. 2B. (H, I) Full-length gels of Fig. 2C.

Suppl. Fig. 8. Full-length gels of Figure 3

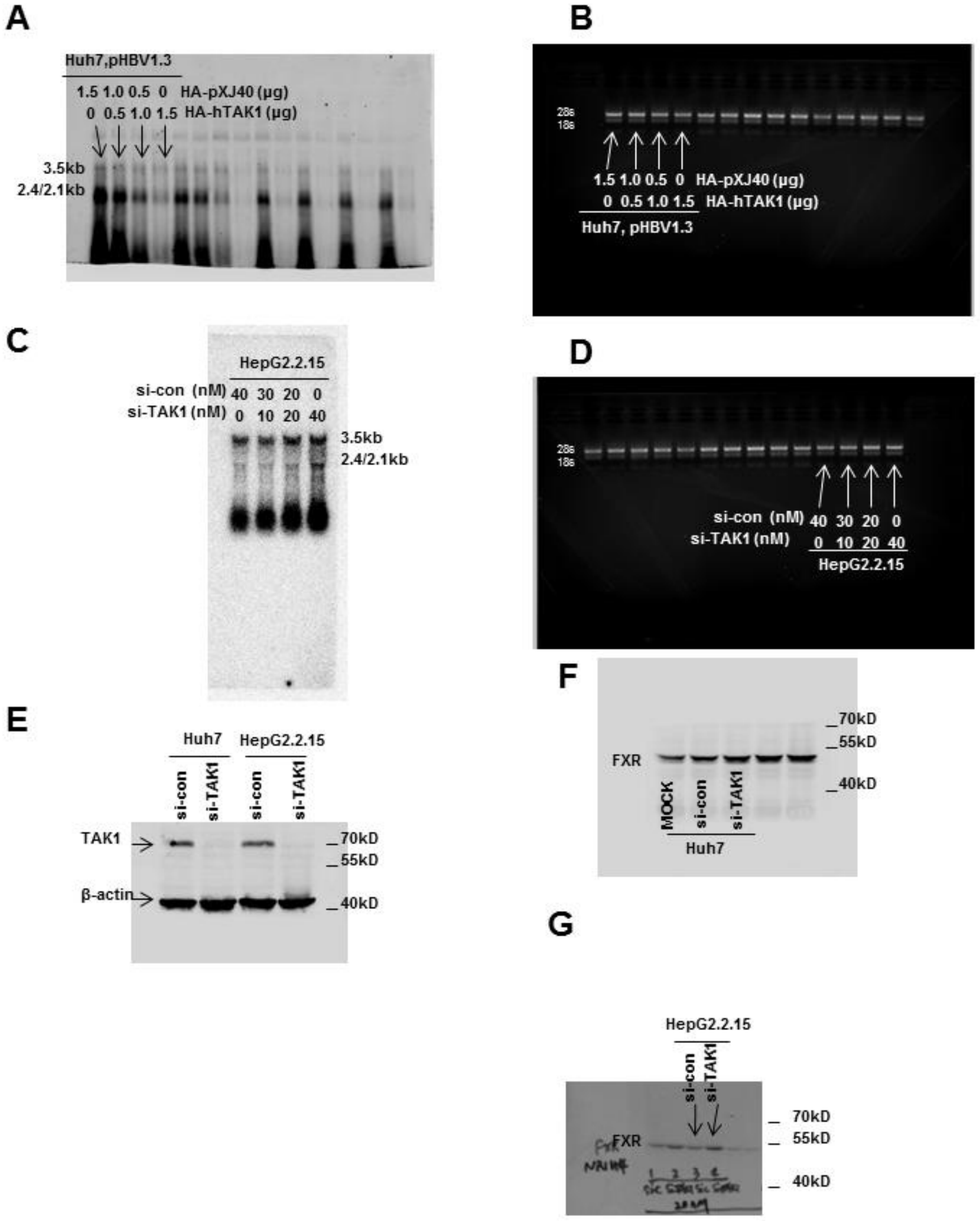

(A, B) Full-length gels of Fig. 3A. (C, D) Full-length gels of Fig. 3C. (E, F, G) Full-length gels of Fig. 3F.

Suppl. Fig. 9. Full-length gels of Figure 4

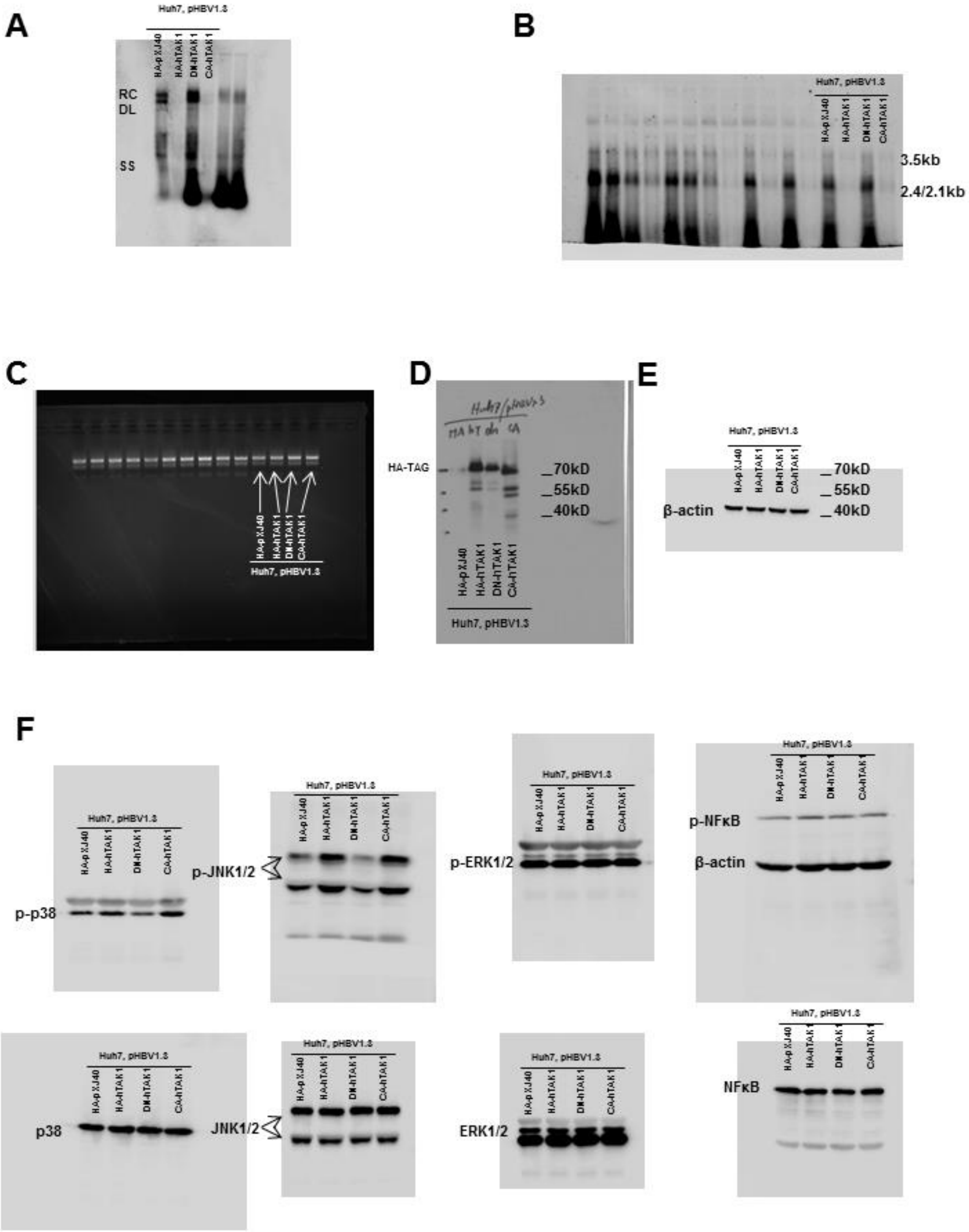

(A, B, C, D, E) Full-length gels of Fig. 4A. (F) Full-length gels of Fig. 4B.

Suppl. Fig. 10. Full-length gels of Figure 5

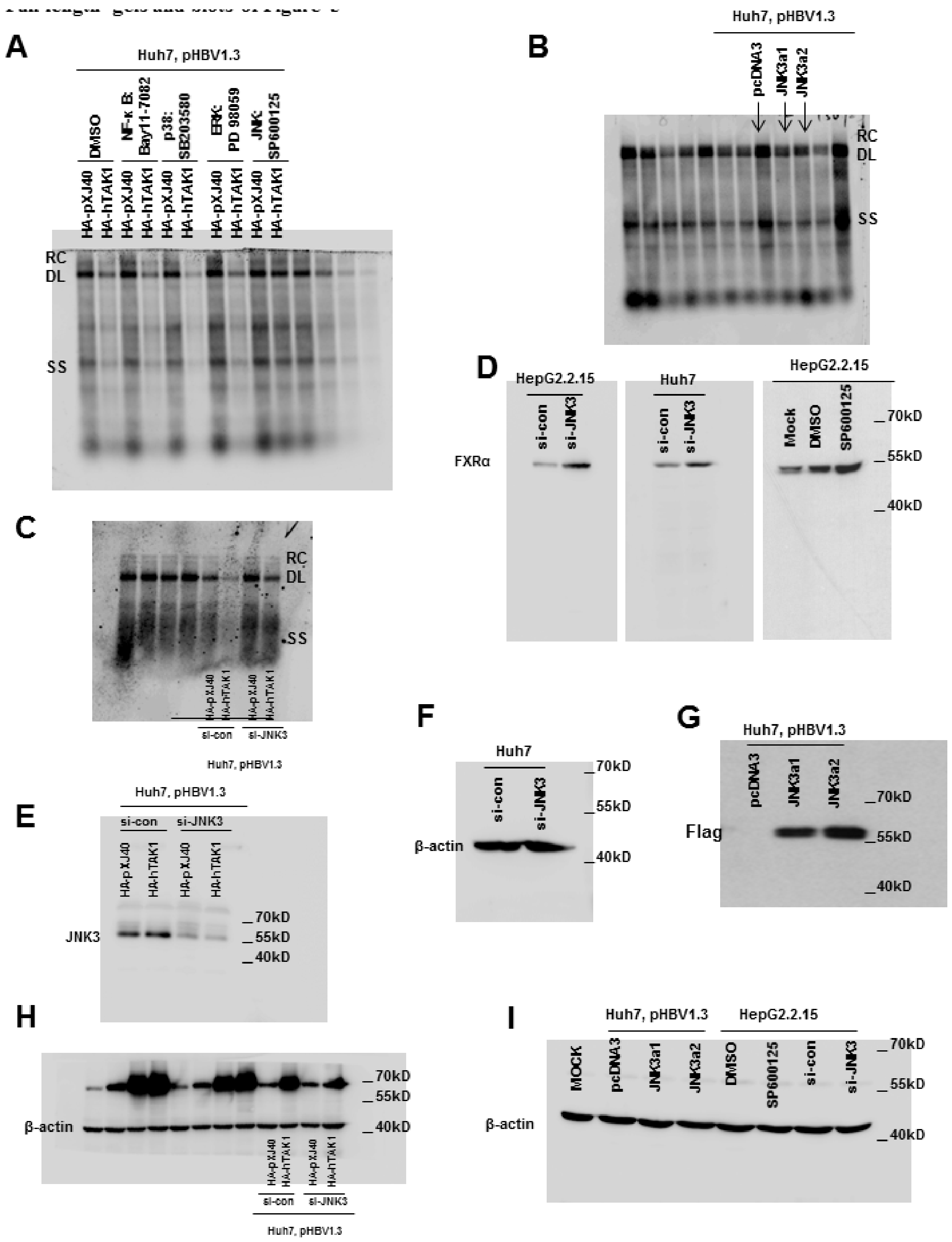

(A) Full-length gels of Fig. 5A. (B, G, I) Full-length gels of Fig. 5B. (C, E, H) Full-length gels of Fig. 5C. (D, F, I) Full-length gels of Fig. 5D.

Suppl. Fig. 11. Full-length gels of Figure 6

A

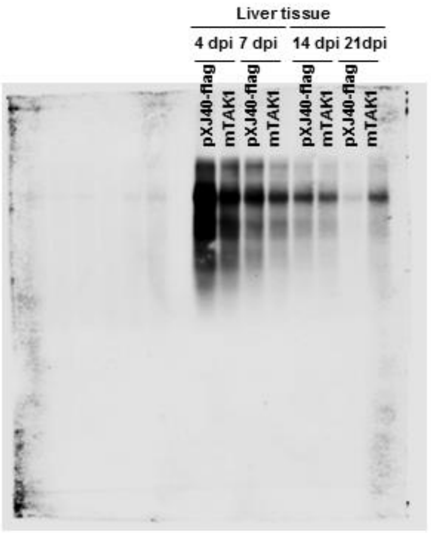

(A) Full-length gels of Fig. 5A. (B, G, I) Full-length gels of Fig. 5B. (C, E, H) Full-length gels of Fig. 5C. (D, F, I) Full-length gels of Fig. 5D.
